# Expoldb: expression linked polymorphism database with inbuilt tools for analysis of expression and simple repeats

**DOI:** 10.1186/1471-2164-7-258

**Published:** 2006-10-13

**Authors:** Vineet K Sharma, Anu Sharma, Naveen Kumar, Mamta Khandelwal, Kiran Kumar Mandapati, Shirley Horn-Saban, Liora Strichman-Almashanu, Doron Lancet, Samir K Brahmachari, Srinivasan Ramachandran

**Affiliations:** 1 G.N. Ramachandran Knowledge Centre for Genome Informatics, Institute of Genomics and Integrative Biology, Mall Road, Delhi 110 007, India; 2 Functional Genomics Unit, Institute of Genomics and Integrative Biology, Mall Road, Delhi 110 007, India; 3 Microarray facility, Department of Biological Services, Weizmann Institute of Science, Rehovot 76100, Israel; 4 Department of Molecular Genetics and Crown Human Genome Center, Weizmann Institute of Science, Rehovot 76100, Israel

## Abstract

**Background:**

Quantitative variation in gene expression has been proposed to underlie phenotypic variation among human individuals. A facilitating step towards understanding the basis for gene expression variability is associating genome wide transcription patterns with potential *cis *modifiers of gene expression.

**Description:**

EXPOLDB, a novel Database, is a new effort addressing this need by providing information on gene expression levels variability across individuals, as well as the presence and features of potentially polymorphic (TG/CA)_n _repeats. EXPOLDB thus enables associating transcription levels with the presence and length of (TG/CA)_n _repeats. One of the unique features of this database is the display of expression data for 5 pairs of monozygotic twins, which allows identification of genes whose variability in expression, are influenced by non-genetic factors including environment. In addition to queries by gene name, EXPOLDB allows for queries by a pathway name. Users can also upload their list of HGNC (HUGO (The Human Genome Organisation) Gene Nomenclature Committee) symbols for interrogating expression patterns. The online application 'SimRep' can be used to find simple repeats in a given nucleotide sequence. To help illustrate primary applications, case examples of Housekeeping genes and the *RUNX *gene family, as well as one example of glycolytic pathway genes are provided.

**Conclusion:**

The uniqueness of EXPOLDB is in facilitating the association of genome wide transcription variations with the presence and type of polymorphic repeats while offering the feature for identifying genes whose expression variability are influenced by non genetic factors including environment. In addition, the database allows comprehensive querying including functional information on biochemical pathways of the human genes.

EXPOLDB can be accessed at

## Background

Functional genomics in the post human genome sequencing era is greatly facilitated by correlating expression data with sequences of potential regulatory elements. The primary repositories of gene expression data such as Gene Expression Omnibus (GEO) [1], UniGene [1], Gene Expression Database (GXD) [2], and Gene Expression Atlas (GNF) [3] provide useful information on gene expression obtained from microarrays and other techniques, however, they provide limited information on the role of genetic elements that can potentially modulate gene expression. Thus, there is a need for databases integrating gene expression information with sequence information of potential genetic regulators with propensity for exhibiting sequence variability.

One such potential regulator is the dinucleotide repeat (TG/CA)_n_. The (TG/CA)_n _repeats are widely distributed, considered to be *cis *regulators of transcription, and above 12 repeat units tend to be polymorphic [4-6]. Segments of DNA consisting of (TG/CA)_n _repeats (with n ≥ 23), display, under conditions close to physiological, the propensity to adopt a Z-form [7-9], a conformation which affects the movement of the RNA polymerase [10], and binding of transcriptional factors [11]. In addition, these repeats have been observed to be associated with recombination sites [12] and mRNA splicing [13], which elect them as functional elements in humans [14].

The (TG/CA)_n _repeats can be divided into three length categories, based on their biological properties [14]. Type I repeats (6 ≤ n < 12) have very low propensity for polymorphism [15]. Type II repeats (12 ≤ n < 23) are likely polymorphic, as more than 93% of the (CA)_n _repeats of n ≥ 12 units were found to display length polymorphism and act as *cis *regulators of transcription [4]. Type III repeats (n ≥ 23) were shown to have a propensity to adopt conformations such as Z DNA [7-10], and to be associated with recombination sites [12]. In general, (TG/CA)_n _repeats of n ≥ 12 units exert a down regulatory effect on transcription, which is positively correlated with the length of repeats [16]. A few examples of genes, whose transcription levels were shown to be modulated by (TG/CA)_n _repeats, are human *HSD11B2 *[16], *MMP-9 *[18], *IFN-γ *[19], *EGFR *[20], and housekeeping genes [22] and others such as rat *α-lactalbumin *[9], *prolactin *[17], and *nucleolin *[11], and tilapia *prolactin-1*[21]. These repeats also exhibit preference for binding to nuclear factors in some instances [24] and stimulate mRNA splicing [13].

In this work, we report the construction of EXPOLDB (EXpression linked POLymorphism DataBase), a novel database focusing on the effect of (TG/CA)_n _repeats on transcription level and variation between individuals. In this first release, EXPOLDB was constructed using expression information from our GeneChip experiments [25] using novel analysis tools.

## Construction and content

### Gene expression data

All data in EXPOLDB resides locally and are retrievable conforming to 'open access'. In this first release, we used GeneChip (HG-U95Av2 arrays, Affymetrix) expression data of blood leukocytes obtained from 13 normal healthy human individuals including 5 pairs of monozygotic twins (GEO series accession No. GSE928) [25].

Mean expression value for each gene was computed from the log_10 _transformed 'signal values' with 'P' (Present) calls. The coefficient of variation (CV) was computed as SD/Mean where SD is the standard deviation. 'Signal log ratio' is the difference in expression level for a transcript between two experiments, and is computed by Affymetrix Microarray Suite Software MAS 5.0. Differentially expressed genes in pair-wise comparisons were identified as those with a signal log ratio > 1.585, after considering the experimental noise [25].

### Sequence retrieval and mapping of (TG/CA)_n _repeats

Genomic sequences of human genes (Build 35) were retrieved in GenBank format from Entrez Gene [1] and parsed to obtain the exon and intron information and the gene sequence. Identification of uninterrupted Type I, Type II and Type III intragenic (TG/CA)_n _repeats was carried out using the Perl script 'SimRep' [14,22]. The genomic region between the most upstream transcript start and the most downstream transcript end of a gene, with the addition of 1 kb 5' of upstream flanking region, was scanned for repeats. The positions of the repeats were noted and displayed with respect to gene structure (exons and introns). For genes with reported alternative spliced variants, the repeats distribution is displayed with respect to the exon-intron structure of the gene corresponding to each individual splice variant. Information on known polymorphic repeats was obtained from CEPH database [26] and mapped to genes using UniSTS [1]. *Alu *repeats were mapped using RepeatMasker [27].

### Expression and functional information from other sources

Information about genes expressed in Blood was derived from Expressed Sequence Tags (ESTs), retrieved from the UniGene database (Build 160) [1]. Genes were classified as highly expressed according to a previously defined criteria (H; >0.0363% of the total detected transcription), moderately expressed (M; from 0.0363% to 0.0121%), or weakly expressed (W; <0.0121%) genes [28]. The dataset of human housekeeping genes was obtained from Eisenberg and Levanon [29]. Pathway information on genes was obtained from NetAffx (version 23^rd ^June 2004) [30].

### Statistical Analysis

Statistical tests were carried out using the 'Statistical Pages' [31]. Differences in expression levels between genes with intragenic (TG/CA)_n _repeats (only Type II and Type III were considered – based on their current experimental evidence as *cis *regulators of transcription with propensity for polymorphism) and genes without repeats (n < 6 units) were evaluated using *t-test*.

### Database and Web interface

The back end data was prepared in MS Access 2000 (Microsoft Corporation Inc., USA). Server side scripting was prepared using ASP (Active Server Pages, Version 3.0), PHP (PHP: Hypertext Preprocessor, version 5.0) and Perl (Practical Extraction and Report Language, version 5.8.1). The client side scripting was prepared using JavaScript and HTML (Hyper Text Markup Language, version 4.0). Internet Information Server (IIS, version 6.0) was used as Web server.

## Utility

**Figure 1:**
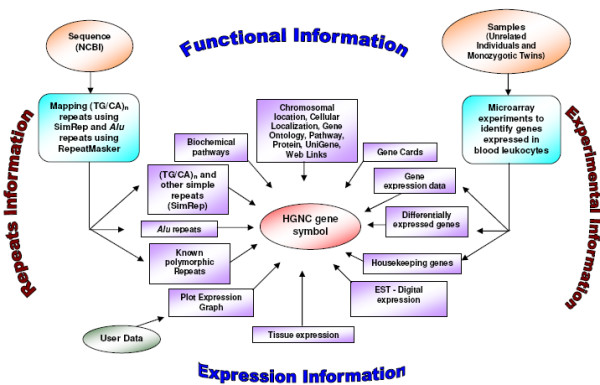
Tetrapodic layout of EXPOLDB: The 4 domains of layout are shown in bold face type. Note that all attributes of a gene are singularly linked to its official HGNC symbol serving as the primary key

The tetrapodic layout of EXPOLDB is shown in Figure 1. All attributes of a gene, are singularly linked to its official HGNC symbol serving as the primary key. A brief description of the potential uses of EXPOLDB is presented below.

### Examining gene expression and variability

EXPOLDB houses gene expression information from monozygotic twins and other unrelated individuals. Queries enable retrieving information about genes expressed in blood, or genes exhibiting inter-individual expression variability. These query pages can be accessed through 'Query EXPOLDB'. Wild cards (*) and multiple keywords can be used with Boolean operators. Queries can be limited by different attributes, such as Chromosome number, HGNC gene symbol, gene function, UniGene ID, accession number of known polymorphic repeats, biochemical pathway, and the range of expression variability. Two indices are provided to assess variation in gene expression: 'coefficient of variation' (CV), and 'signal log ratio'. CV is provided with the 'Expression in Blood" query page, and indicates the overall variability in a set of individuals. We have presented one application of CV, to identify 'control' or 'reference' housekeeping genes whose expression is most constant across all individuals regardless of their genetic relationship [32]. On the other hand, 'signal log ratio' is provided with the "Differential Expression" query page, and since it is generally used for pair-wise comparisons it is more suitable to assess differential expression between monozygotic twins. Discordance in expression in this case could indicate the lack of genetic effect, and potential involvement of environmental factors. The mathematical relationship between the two measures has not been worked out yet in the literature and therefore, users are advised to exercise caution while using the two measures. In principle, the use of a given metric is guided by the biological question at hand.

The query for differentially expressed genes offers three limited sets, in addition to searching all genes: (1) Genes differentially expressed in monozygotic twins, which are less likely to be influenced by genetic factors, (2) Genes differentially expressed in unrelated, age matched (20–23 yr) female individuals but not in monozygotic twins (including all possible pair-wise comparisons), these are more likely to be affected by genetic factors, and (3) Differentially expressed housekeeping genes (in all pair-wise comparisons including monozygotic twins).

### Querying

Submission of a query produces a 'Results' page listing all the resultant gene matches. The expression of genes in different individuals can be examined visually either singly or collectively as bar charts in the graphic display by selecting the appropriate square boxes placed in front of the listed genes. Detailed information of genes (EXPOLDB profile) can be obtained by clicking on the HGNC gene symbol displayed on the 'Results' page.

### EXPOLDB Profile

The EXPOLDB profile of a gene summarizes the information on the function, expression and repeat content of the gene. The table 'Expression in Blood' provides mean expression, coefficient of variation (CV) and EST based expression status ('H'/'M'/'W' for High/Medium/Weak). The expression levels of a gene in unrelated age and gender matched individuals and in monozygotic twins can be examined visually in the form of bar charts by clicking on the 'Show Expression Graph' button. The table 'Differentially expressed genes' displays information in the two categories 'Unrelated age and gender matched female individuals' and 'Monozygotic Twins'.

### Repeat Table

The table '(TG/CA)_n _Repeats' provides information on (TG/CA)_n _repeats categorized into three types (I, II and III). The lengths and positions of repeats within the gene structure (exons and introns) and in 1 kb upstream flanking region are displayed. Because stretches of repeats separated by short intervals may act in concert to modulate transcription [33], the table also reports (TG/CA)_n _repeats within a range of 50 bp flanking each repeat. The table 'Polymorphic Repeats' provides information on the presence of known polymorphic repeats with their heterozygosity index and the number of alleles in CEPH families [26]. All this information can also be retrieved collectively for multiple genes by using the 'Advanced Query for Retrieving Data for Multiple Genes' option available on the Results page.

Information on other intragenic simple repeats can be probed in the table 'Other simple repeats' by specifying the repeat type, minimum cut-off length to score a repeat and clicking on the button 'Run SimRep'. The table on '*Alu *Repeats' provides information on the total content of intragenic *Alu *repeats. The details specifically for the young and active *Alu *Y repeats can be pulled out by clicking on the button '*Alu *Y'. Other related information on gene function and expression can be accessed using the links provided to the publicly available databases such as KEGG [34], GeneCards [35], GDB [36], UCSC Golden Path [37], Ensembl [38], PubMed [1], GXD [2], HuGEIndex [39] and GNF [3].

### SimRep – An online application to identify simple repeats

'SimRep' is an online application to identify dinucleotide and other microsatellite repeats in a given nucleotide sequence including all available human gene sequences. Users can search either for a dinucleotide repeat by selecting it from the pull down menu or for a specific microsatellite repeat of their choice by entering the pattern to be searched. Patterns can be specified using the standard four base symbols A, T, G and C, as well as other symbols recommended by IUPAC (International Union of Pure and Applied Chemistry). The minimum length for scoring a repeat can be specified in the field 'Enter Cut-Off'. SimRep reports the length and location of the repeats or patterns in the given sequence in the form of a table. The positions of repeats either in forward strand (+) or in reverse strand (-) are reported with respect to the forward strand only as per the convention followed by genome sequence annotation groups. In the case of palindromic dinucleotide repeats such as GC, AT only one strand is reported.

### Examining gene expression and variability in Biochemical Pathways

**Figure 2:**
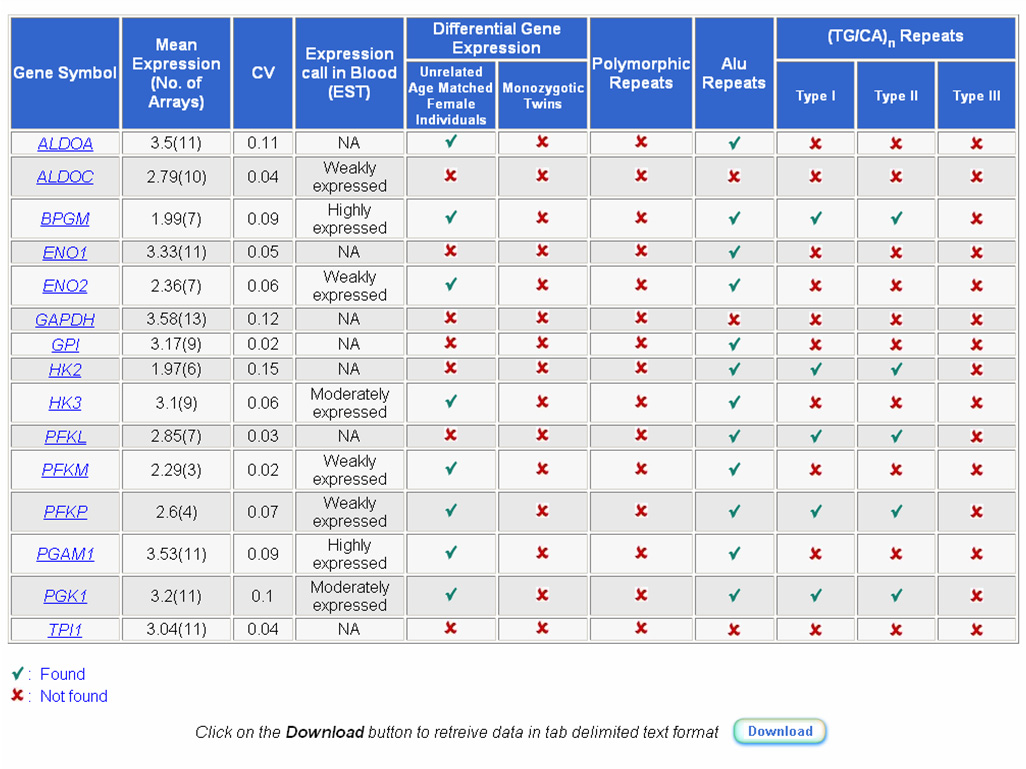
A summary of the expression patterns and repeat distribution in genes of the glycolytic pathway.

With the present focus of biology shifting towards adopting a systemic approach to understand the complexity of human biology, biochemical pathways have become a focus of investigations. EXPOLDB offers this facility by providing information on gene expression and its variability in 134 biochemical pathways (from the KEGG and GenMAPP databases) as organized by NetAffx [30]. Information on expression patterns including expression status, variability between monozygotic twins and between unrelated individuals (age and gender matched), repeats, polymorphic markers and functions can be queried for the genes involved in a defined pathway. For example, in the glycolytic pathway (Figure 2), none of the genes were differentially expressed in the monozygotic twin pairs, indicating that if expression variability is found for genes in this pathway it is likely not determined by environmental factors. Only 5 genes (*BPGM*, *HK2*, *PFKL*, *PFKP*, and *PGK1*) out of 15 genes (including isoforms) contained Type I and II repeats. 9 out of 15 genes (including isoforms) had low CVs (≤ 0.08) across all individuals and 8 were differentially expressed among unrelated individuals (age and gender matched). Among the 5 genes with Type I and II repeats, 3 (*BPGM*, *HK2*, *PGK1*) had CVs ≥ 0.8 and 3 (*BPGM*, *PFKP*, *PGK1*) were differentially expressed. These observations support the traditional practice of using the genes of the glycolytic pathway as 'controls' or as 'reference' genes in gene expression studies. Three genes *ALDOC*, *ENO1 *and *GPI *showed no variation in expression between any two pairs of individuals including monozygotic twins, contained no repeats and had low CVs (<0.08) and therefore are devoid of potential factors that causes expression variation. If verified independently, these genes could be used as 'controls' or 'reference' genes in mRNA quantitation experiments.

### Correlating genome wide expression with the incidence of (TG/CA)_n _repeats

Eukaryotic transcription is inherently complex and involves interaction of large numbers of proteins [40]. Therefore, examining the role of regulatory elements in gene expression regulation requires a set of genes with either a common organization of promoter, upstream elements, CpG islands etc., or with common expression profiles emanating from their clustered localization in coordinately regulated genomic regions [41]. Examples for gene sets conforming to these specifications are housekeeping genes, and genes belonging to the same family with similar architecture of regulatory elements such as the *RUNX *gene family [42].

#### Housekeeping genes

Housekeeping genes are expressed constitutively in all tissues to maintain cellular functions, and their expression pattern is less likely to be affected by variations in tissue specific factors, polymorphism in chromatin structure across different individuals, or experimental artifacts such as the number of different cell types in blood samples (if similar quantities of total RNA is taken) [39,41]. In the human housekeeping genes, the mean expression of genes without repeats (n < 6 units) was observed to be significantly higher than the mean expression of genes containing Type II and Type III (TG/CA)_n _repeats (*t-test*, df = 455, *P *< 0.006) suggesting the down modulatory role of these repeats to be in conformity with previous observations [9,14-23].

#### The RUNX family

Similar analysis was carried out with *RUNX *family. The mammalian RUNX genes comprise a small family of three genes *RUNX1*, *RUNX2 *and *RUNX3 *containing the 'runt domain' (RD), that act as master regulators of gene expression in major developmental pathways [42,43]. Sequence analysis suggests that *RUNX3 *is the evolutionary founder of the mammalian *RUNX *family [43], and there exists extensive structural similarities between the three mammalian *RUNX *genes. Thus, the *RUNX *family provides a set of genes with similar architecture to investigate the effects of (TG/CA)_n _repeats in expression.

The records for *RUNX1 *and *RUNX3 *were retrieved (*RUNX2 *was not found to be expressed in blood). The gene *RUNX1 *has several Type II (TG/CA)_n _repeats: (CA)_17_, (CA)_22_, (TG)_12_, (TG)_13_, (TG)_14_, (TG)_21_, (TG)_23_, (TG)_24 _and (CA)_22 _in introns and one interrupted (TG)_7_-CG-(TG)_9 _repeat in exon 8, whereas *RUNX3 *is devoid of these repeats (n < 6 units). The mean expression of *RUNX3 *is significantly higher than the mean expression of *RUNX1 *(*t-test*, df = 13, *P *< 0.0002). These results corroborate with the common trend of down regulatory role of repeats as observed earlier [9,14-23].

### Literature Resource

We have compiled a useful list of publications of several studies from the perspective of (TG/CA)_n _repeats as *cis *modulators of gene expression and other upcoming multifaceted roles of these repeats [23]. This list is likely to grow with the availability of more information and at present provides a useful wealth of information on the multifaceted roles of these repeats.

## Discussion

### Similar Databases

To our knowledge, EXPOLDB is the first systematic attempt to correlate gene expression and its variability with the presence and type of (TG/CA)_n _repeats. Other available databases focus singly on either gene expression or repetitive sequences.

### Unique features of EXPOLDB

EXPOLDB is constructed for facilitating examination of the effect of repetitive elements in *cis *on expression variability. In addition, it allows distinguishing between genetic factors and other factors influencing expression levels (such as environment), by comparing expression between monozygotic twins. In particular, this data could be used as a sieve while identifying genes whose expression varies primarily due to genetic factors. Further, the variability in expression and the repeat content can be examined and correlated using either a gene centric or a pathway centric approach. Graphic display of expression values in the form of bar charts aids visual comparisons and stimulates novel questions. The tool SimRep can be used to identify other dinucleotide and user specified simple repeats in recent build (build 35) of human genes sequences and in a given nucleotide sequence.

### Limitations

At present, as per global status, there is limited data on monozygotic twins and on the polymorphic status of several repeats and on gene expression data from different populations. Our efforts in constructing EXPOLDB are likely to stimulate and facilitate investigations on this aspect of variability in gene expression. We envisage that the emerging role of (TG/CA)_n _repeats as 'functional elements' [4-23] and the global efforts on generating expression data are likely to result in the growth and use of this database.

## Conclusion

We envision that our effort to organize the gene expression data and the variability contained in it from the perspective of simple repeats as *cis *regulators of transcription could enhance other efforts in this subject and could serve as a seed database by offering genome wide expression data with facility to correlate genetic information for Systems Biology projects.

## Availability and requirements

EXPOLDB is accessible at  requires Explorer version 5.5 or above, FireFox version 1.0.4 or above.

## List of abbreviations

EXPOLDB: Expression Linked Polymorphism database, CV: Coefficient of Variation, SD: Standard deviation, HGNC: HUGO Gene Nomenclature Committee

## Authors' contributions

This project symbolizes Indo-Israel friendship with mutually benefiting interests. All authors have contributed together towards this goal. VKS carried out a major part of the work including writing of computer programs, planning artistic GUI, downloading data from NCBI, analysis of microarray and sequence data and wrote the manuscript, AS helped in carrying out the microarray experiments and analysis of microarray data, NK and MK helped in software coding web enablement, SHS helped in carrying out the microarray experiments, LSA and DL offered constructive scientific criticisms with focus on providing benefits from user view point, SKB provided scientific suggestions particularly on twins during the work. SR is the group leader, working in many arms of the project including experiments, provided scientific suggestions and criticisms for improving the database, guided in statistical analysis, conforming to ethical principles, critical examination, presentation and manuscript preparation.
